# Gleanings in Science and Medicine

**Published:** 1893-08-19

**Authors:** 


### Gleanings in Science and Medicine.

A State in the South-Eastern part of Africa has set the example of combining the double cure, the spiritual and the physical, in one person; this finds its representative in no less a person than that of the Rev. William E. Smyth, M.D., who has been appointed to the newly-inaugurated medical bishopric by the authorities of the Anglican Church.

Reading aloud is an art but little understood at present amongst the cultivated and highly educated in the land. There is always too much effort made to be heard or too little, the voice is seldom allowed to be natural, and with any sense of strain all enjoyment on the part of the listener ceases. The real secret of the art is to use the mid-level or natural tone of the voice, but curiously enough this simple expedient is the very last which seems to present itself to the brain of the amateur reader.

The Metropolitan Public Gardens Association in their zeal for maintaining open spaces have originated the somewhat incongruous scheme of utilising old City churchyards as recreation grounds for the people. At first it strikes one as pathetic that the last quiet resting place of one generation should be the playing ground of another; but in this, as in many other cases, sentiment and hygiene do not go together hand in hand, on the threshold of the latter, the former must be prepared to surrender its claims, and now in this scheme just set forth the churchyards of St. Olave, in Silver Street, City, and St. Katherine Coleman, in Fenchurch Street, will shortly open their gates in favour of the living.

One of the important questions which has been treated by the Tuberculosis Congress at Paris has been the obligatory cremation of the remains of persons who die of consumption. It is stated that bacilli which exist in those bodies are brought in dry weather to the surface of the ground by earth worms. Two well-known Lyons doctors quoted cases, the results of their own experiments in favour of the fact, that the disease has clearly been propagated through this means. The method employed in the experiments to prove this was firstly to place the sputa of consumptives in pots prepared to receive it; then to examine earth worms which were found to be tubercular (as also they were found to communicate the disease to other animals hitherto unaffected). In the face of this experience some doctors are now demanding compulsory cremation as one means of guarding against a wider spread of consumption.

**At the Hornsey Sanitary Museum an interesting experiment with regard to sewer ventilation is being carried on. Of the many shafts thus investigated, the straight perpendicular one has been found the most efficacious. The ventilators are kept constantly at work, a fine clockwork apparatus registering the amount of air that passes through each of them. There are on view, to illustrate former "perfect sanitary arrangements," a stone pipe, which is filled with deposits of hard stone, of oval formation through the passing of warm water, as, indeed, also a lead soil pipe is exhibited, which is a specimen of inside-house drainage, and which is eaten through and through by gases which could not escape owing to no ventilation being provided.**

At the last meeting of the Willesden Local Board Dr. Skinner, the Medical Officer of the Isolation Hospital, wrote stating that in his desire to have the small-pox patients regain strength as speedily as possible, so as to be able to close the tents, he had allowed them ginger beer. These were the only patients who had stimulants, excepting in cases of very severe illness, in which he had been obliged to allow brandy.

Dr. W. B. Johnson read a paper at the recent assembly of the Medical Society of New Jersey on the "Prospective Legislation for the Prevention of Blindness." The ratio of increase of blindness, he pointed out, was greater than that of increase of population. There were ways and means of stopping the rapid spread of ophthalmic contagion, which New York State and other countries had already discovered. The evil was a very serious one, and steps ought to be taken to check its growth. If midwives were held legally responsible to report all cases of ophthalmic affection which came within their dominion, and it were made illegal to suppress the knowledge of contagious diseases of this kind, it would necessarily diminish the increase of the evil, through giving others a chance of avoiding the contagion.

The stamping of Australian meat has, according to a weekly contemporary, a domestic economic side, apart from its more strictly international one. The writer of an article on this subject deals with the serious and substantial arguments in favour of this new regulation in a light and humorous manner and enumerates fresh and unthought of reasons in its favour, arguments, perhaps, of a domestic economic nature, rather than a national value. "Having" (so runs the wording) "helped Mr. Scrimp to the central T for his Sunday dinner, and taken the s, r, and a for herself and the children, she will give Sarah Jane leave to fatten on the capital A of the knuckle end, and require that domestic to produce on Monday the U and the final ALIA, under pain of instant dismissal. If there should be any of those letters missing, she will know at once that unholy appetites have been at work, and can bait her traps for policemen or cousins from the country."

However much sympathy the existence of the workers in coal pits may evoke from us, yet the actual result of the life on the physical welfare of the miners is by no means such as to warrant any serious commiseration at our hands. Indeed, if one may judge from the physique of the underground toilers of the Black Country, they present an appearance more provocative of envy than of pity. Disease is no more demolishing in its raids amongst them, as a class, than it is amongst the agriculturists and labourers. If one can be guided by statistics, the coal-dust atmosphere in which their life is passed is no element of any serious evil to them; it may not be pleasant, but it is not unhealthy, indeed, the actual death-rate of those miners is not abnormally high, even when is included the fatal wholesale disasters which occur from time to time in the pits. However much reason the promoters of the pending coal strikes may have to substantiate their theories, the evil of any extreme insanitary condition of existence to the workers themselves below ground cannot by any fairness be included.

